# A SIMBA CoMICs Initiative to Cocreating and Disseminating Evidence-Based, Peer-Reviewed Short Videos on Social Media: Mixed Methods Prospective Study

**DOI:** 10.2196/52924

**Published:** 2024-10-30

**Authors:** Maiar Elhariry, Kashish Malhotra, Kashish Goyal, Marco Bardus, SIMBA and CoMICs Team, Punith Kempegowda

**Affiliations:** 1 Sandwell General Hospital Sandwell and West NHS Trust Birmingham United Kingdom; 2 Applied Health Sciences, School of Health Sciences College of Medicine and Health University of Birmingham Birmingham United Kingdom; 3 Rama Medical College Hospital and Research Centre Hapur India; 4 Delhi Heart Institute and Multispeciality Hospital Bathinda India; 5 School of Medical Sciences & Research Sharda University Greater Noida India; 6 see Authors' Contributions; 7 Queen Elizabeth Hospital University Hospitals Birmingham NHS Foundation Trust Birmingham United Kingdom

**Keywords:** influencers, social media, public engagement, apps, healthcare, medical students, online medical information, simulation, peer-reviewed information

## Abstract

**Background:**

Social media is a powerful platform for disseminating health information, yet it is often riddled with misinformation. Further, few guidelines exist for producing reliable, peer-reviewed content. This study describes a framework for creating and disseminating evidence-based videos on polycystic ovary syndrome (PCOS) and thyroid conditions to improve health literacy and tackle misinformation.

**Objective:**

The study aims to evaluate the creation, dissemination, and impact of evidence-based, peer-reviewed short videos on PCOS and thyroid disorders across social media. It also explores the experiences of content creators and assesses audience engagement.

**Methods:**

This mixed methods prospective study was conducted between December 2022 and May 2023 and comprised five phases: (1) script generation, (2) video creation, (3) cross-platform publication, (4) process evaluation, and (5) impact evaluation. The SIMBA-CoMICs (Simulation via Instant Messaging for Bedside Application–Combined Medical Information Cines) initiative provides a structured process where medical concepts are simplified and converted to visually engaging videos. The initiative recruited medical students interested in making visually appealing and scientifically accurate videos for social media. The students were then guided to create video scripts based on frequently searched PCOS- and thyroid-related topics. Once experts confirmed the accuracy of the scripts, the medical students produced the videos. The videos were checked by clinical experts and experts with lived experience to ensure clarity and engagement. The SIMBA-CoMICs team then guided the students in editing these videos to fit platform requirements before posting them on TikTok, Instagram, YouTube, and Twitter. Engagement metrics were tracked over 2 months. Content creators were interviewed, and thematic analysis was performed to explore their experiences.

**Results:**

The 20 videos received 718 likes, 120 shares, and 54,686 views across all platforms, with TikTok (19,458 views) and Twitter (19,678 views) being the most popular. Engagement increased significantly, with follower growth ranging from 5% on Twitter to 89% on TikTok. Thematic analysis of interviews with 8 out of 38 participants revealed 4 key themes: views on social media, advice for using social media, reasons for participating, and reflections on the project. Content creators highlighted the advantages of social media, such as large outreach (12 references), convenience (10 references), and accessibility to opportunities (7 references). Participants appreciated the nonrestrictive participation criteria, convenience (8 references), and the ability to record from home using prewritten scripts (6 references). Further recommendations to improve the content creation experience included awareness of audience demographics (9 references), sharing content on multiple platforms (5 references), and collaborating with organizations (3 references).

**Conclusions:**

This study demonstrates the effectiveness of the SIMBA CoMICs initiative in training medical students to create accurate medical information on PCOS and thyroid disorders for social media dissemination. The model offers a scalable solution to combat misinformation and improve health literacy.

## Introduction

In July 2023, there were more than 4.9 billion social media users globally, equating to over 61% of the world’s population [1]. Social media usage has increased by 3.7% in the past year, with 173 million new users (5.5 new users every second). Checking one’s social media profile has become a predominant activity for 9 out of 10 internet users. Furthermore, 7 of the top 10 most popular social media platforms claim over 1 billion monthly active users. These are Facebook (Meta; 2.989 billion), YouTube (Google; 2.537 billion), WhatsApp (Meta) and Instagram (Meta; 2 billion), WeChat Inc or Weixin (Tencent; 1.319 billion), TikTok (ByteDance; 1.081 billion), and Facebook Messenger (Meta; 1.038 billion). In addition, 4 platforms are owned by the same company, Meta (Facebook, Instagram, WhatsApp, and Facebook Messenger). These are followed by Snapchat (Snap Inc, 750 million users), Douyin (ByteDance, 730 million users), and Telegram (Telegram Messenger Inc, 700 million users). Social media reach extends beyond personal interactions on each platform as users adopt multiple platforms. For example, nearly 78% of Facebook users also use Instagram.

GWI’s data from DataReportal reported that 49% of active users aged 16 to 64 (outside China) use social media to keep in touch with friends and family, 37% to fill spare time, 35% to read new stories, 30% to find content (studies and videos). While some platforms are used for passive entertainment (eg, TikTok), Instagram, Facebook, and Snapchat are used for content creation by sharing posts and videos. Given its reach, social media has also emerged as a powerful tool for promoting health and disseminating health-related research findings, surgical education, and medical information [2-4].

However, the role of social media in sharing clinical experiences is complex and carries potential benefits and challenges. Some benefits include rapid and wide dissemination of information at minimal costs to the end user, bridging the gap to health care access, and patient education [5]. However, this unprecedented access to information may also provide a breeding ground for misinformation. This spread of misleading or false information can have dire consequences in health care, where accurate knowledge is crucial for making informed decisions about one’s well-being [6]. This risk comes with the exponential growth of short video platforms such as TikTok, mimicked by Instagram reels, Facebook stories, and YouTube shorts, whose algorithms tend to propose similar content based on the users’ histories and preferences. Short video platforms are echo chambers [7] that reinforce beliefs, prejudices, fake news, and misinformation. There is a need to address this by producing evidence-based content to ensure the dissemination of accurate information without bias [7,8].

Considering the negative consequences of misinformation, in the last 2 years, the World Health Organization partnered with major technology companies that have leverage on major social networking sites such as Alphabet (Google and YouTube) [9] and Meta (Facebook, Instagram, and WhatsApp) [10] to minimize misinformation. Solutions included semiautomated flagging, labeling, or removing content that violates community guidelines and misinformation policies [11]. Google and YouTube have recently invested US $13.2 million in the International Fact-Checking Network [12] to enhance misinformation response. Video creation platforms, such as YouTube, Twitch, or TikTok, have their community guidelines, which try to regulate the content produced. Nevertheless, there is little to no guidance on creating content before it is uploaded on social media platforms.

Regarding medical or health-related content, social media platforms generally include content that rarely reflects clinical guidelines (eg, low back pain and laparoscopic hysterectomy) [13,14]. Many studies report methods for evaluating medical content on social media, specifically YouTube [15], but few describe medical education videos’ development, implementation, and evaluation.

Since, to the best of our knowledge, there are no specific international guidelines to create evidence-based medical and health content related to polycystic ovary syndrome (PCOS) and thyroid disorders, an international medical education initiative was launched to create evidence-based and peer-reviewed bite-sized videos on various medical conditions in collaboration with various patient support groups [16-19]. The initiative named “SIMBA CoMICs” (Simulation via Instant Messaging for Bedside Application–Combined Medical Information Cines) involves medical students, junior doctors, and patient groups who collaborate to create bite-sized videos for different social media platforms. Combined Medical Information Cines (CoMICs) initiative is a novel approach merging intricate medical concepts with illustrative graphics, presented in video format for swift assimilation. Each CoMIC video meticulously portrays distinct medical conditions, encompassing their presentations, diagnostic tools, subsequent treatment options, and recommended follow-up measures. The content for each presentation aligns with national and international guidelines and undergoes rigorous evaluation by leading experts in the corresponding medical domain.

This study describes generating, creating, disseminating, and evaluating evidence-based, peer-reviewed, short social media videos about PCOS and thyroid diseases. The project followed a collaborative approach with people living with these conditions and health care professionals with a special interest in these conditions. We analyzed the project outreach and audience engagement on social media (specifically TikTok, Instagram reels, YouTube shorts, and Twitter). Furthermore, we gathered participant experience for effective engagement and evidence synthesis. This examination of public discourse through social media platforms provides insights into disseminating and perpetuating viewpoints regarding thyroid conditions and PCOS.

## Methods

The SIMBA-CoMICs (Simulation via Instant Messaging for Bedside Application–Combined Medical Information Cines) initiative provides a structured framework for simplifying medical concepts and converting them into visually engaging, evidence-based videos. The initiative actively recruited medical students and resident doctors, who were guided through the script creation and video production process. The SIMBA-CoMICs initiative emphasised on collaboration among health care professionals, content creators, and individuals with lived experience of PCOS and thyroid conditions to ensure that the final content was scientifically accurate and relatable to the public. This structured approach ensured consistency across all phases of the project, from initial script development to final video dissemination on social media platforms.

### Design

**Figure 1:**
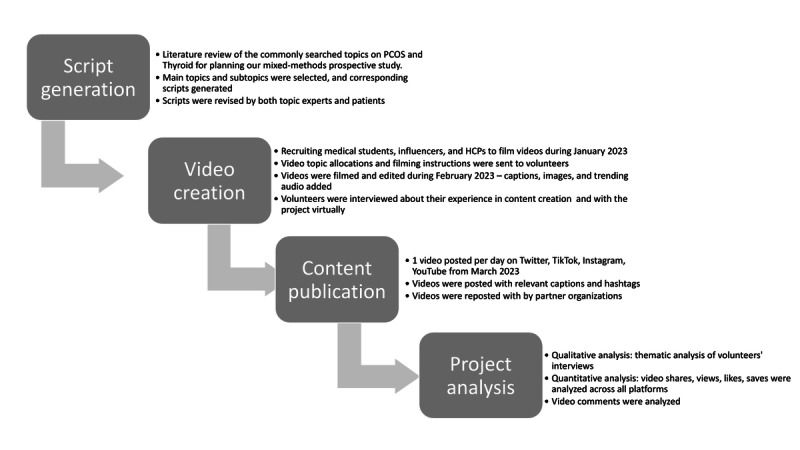
Overview of the main steps to complete this project. HCP: health care professionals; PCOS: polycystic ovary syndrome.

This mixed methods prospective study was conducted between December 2022 and May 2023 and consisted of 5 phases: script generation, video creation, cross-platform publication, process, and impact evaluation (Figure 1). This study was inspired by the “Knowledge-to-action (KTA)” framework, whereby the content of the videos is based on scientific evidence synthesis, translation, and validation through expert consultation and patient engagement [20]. The content is then produced, and its fruition is monitored, evaluated, and critically appraised for improvement and for generating recommendations.

### Script Generation

A literature search of the frequently asked questions in PCOS and thyroid conditions was conducted across various databases, including PubMed, Cochrane Library, and Embase. Key MeSH (Medical Subject Headings) terms, including “frequent,” “questions,” “popular,” “topics,” “Thyroid,” “conditions,” “PCOS,” and “Polycystic Ovary Syndrome,” were explored across all databases in this scoping review. Based on this, a list of the most popular topics was created. The experts and representatives of patient support groups reviewed the topics independently to arrive at 5 main topics for both PCOS and thyroid. Each topic was subdivided into 3-5 subtopics. We then created corresponding scripts and guidance on when and how to seek medical intervention or advice. The scripts’ scientific accuracy and ease of understandability were checked by 2 early career researchers, a consultant endocrinologist, and members of patient support groups to ensure they align with the relevant international guidelines.

### Video Creation

We invited medical students and junior doctors globally to contribute to this project by advertising the role of video creation on our social media handles (Twitter: SIMBAsimulation, TikTok: simbacomic, Instagram: simba.comic, YouTube shorts: @simbasimulation8047). Medical students at the University of Birmingham and other authors’ institutes were also invited by email to express interest in creating these videos. Similar invites were sent to members of UK-based and international patient support groups supported by partnering institutions (PCOS Vitality and British Thyroid Foundation). Patient support groups were invited to contribute to the videos. Participants were incentivized by certificates acknowledging their contribution to this project. The invitations included a brief role description and instructions on the format that the volunteers will be asked to film themselves.

Upon expressing interest, each volunteer received an email with the script of the content they were allocated in the video series. The emails also included a sample video for participants to visualize the expected product. Participants were requested to film in portrait orientations in keeping with the main format of short videos on TikTok, Instagram reels, YouTube shorts, and Twitter. In addition, we advised participants to keep the videos under 30 seconds to match the average video duration on social media [21]. Participants who submitted a video were invited to create more videos within an agreed set of personalized deadlines. A total of 5 volunteers agreed to this. After we received all the videos, we added the transcripts to the video as captions, one line at a time, and highlighted keywords, with the relevant pictures and emojis to help the audience better understand and interpret the videos. We edited the videos where necessary to ensure they did not exceed 30 seconds. The finalized videos were shared with early-career endocrinologists and members of patient support groups, who reviewed them and helped finalize the transcripts. Further changes were made to the videos to ensure accuracy and acceptability.

### Content Publication

The finalized videos were uploaded to TikTok to be edited using the app’s video editing features, followed by sharing them as Instagram reels, YouTube shorts, and Twitter as a series of video posts. A target minimum of one video was posted every day between March 2, 2023, and March 26, 2023, with a 1-day break after each video series. The videos were posted with their subheading as the video’s public title, and relevant, popular hashtags were included for each video to stimulate the social media algorithm and help redirect the videos to interested audiences. Volunteers were tagged in the videos they filmed if they had an account on the social media platform used after their consent.

### Process Evaluation: Participants’ Experience

We invited our content creators to share their views about the project in a semistructured online interview. The interview specifically aimed to explore their experiences, motivation to participate, and interest in developing similar videos for other subjects. Participants were reassured that their responses would be anonymized and would not impact whether they received a certificate for contributing to this project. These interviews were conducted on Zoom (Zoom Video Communications, Inc) and lasted approximately 10 minutes. Each interview was recorded after consent, and participants were allowed to have their cameras switched off during the meeting. All study data were stored in a password-protected folder, with only the study team having access to it. All questions were asked to each participant following the set order, as indicated in the interview guide provided in Multimedia Appendix 1. Interviews with the participants were anonymized and transcribed verbatim. Furthermore, 2 independent authors did the coding of interview transcripts using NVivo (version 12.0). The codes were combined to identify themes using thematic inductive analysis [22,23]. The research team discussed the codes and agreed on the thematic structure proposed.

### Impact Evaluation: Quantitative and Thematic Analysis

We evaluated the impact of the produced videos using a combination of quantitative and qualitative data. Quantitative data analyses were based on video analytics on each platform, including the total number of views, the highest number of views on a video, total shares, total number of saved videos, total likes, and change in the number of followers or subscribers were extracted along with other similar variables across all platforms after a month of publishing the video. Considering the proprietary algorithms, recommendations, and search engines followed by each platform, comparative statistical tests were not run across platforms. Qualitative data was based on a content analysis of the comments posted under each video to understand the viewers’ perspectives. Comments were inductively coded.

### Ethical Considerations

No patient data were collected, and this study was approved by the ethics committee of Delhi Heart Institute and Multispecialty Hospital (DHIMH/IEC/2023-008). Informed consent was taken from each participant, and participation was voluntary.

## Results

### Script Generation

The topics and subtopics of the videos we generated through our literature searches and validated through expert consultations are presented in Textbox 1.

**Outline of topics and subtopics of the videos.**

**Thyroid nodules:**

- Definition and differentials
- Red flag symptoms
- Investigations
- Management

**Hypothyroidism:**

- Symptoms and signs
- Investigations
- Risk factors
- Management

**Diagnosing polycystic ovary syndrome (PCOS):**

- Diagnostic criteria
- Symptoms
- Associated complications
- Risk factors
- Emotional well-being

**PCOS implications and associations:**

- Impact on menstruation
- Impact on pregnancy and its likelihood
- The link between PCOS and thyroid

**Thyroid and pregnancy:**

- Conception
- Effect of hypothyroidism
- Effect of hyperthyroidism
- Impact on infants
- Breastfeeding

### Study Participants and Content Created

We recruited 38 content creators, mostly students (33/38, 87%), 2 people with 1 or more of the conditions, and 2 social media influencers. In total, 11 students and 1 self-identified influencer created 21 videos.

### Process Evaluation: Participants’ Experience

Out of the 12 volunteers who filmed the videos, 8 (67%) completed an interview on their experiences and views on the project. Thematic analysis of the anonymized interview transcripts yielded 4 main themes, that are, views on social media, advice when using social media, the reason for taking part in this project, and thoughts on this project (Table 1).

**Table 1:** Outline of central themes and subthemes from the thematic analysis of participant interviews.

Theme	Subthemes
Views on social media	Disadvantages, advantages, and uses of social media.
Advice on making the most of social media	Factors impacting public engagement, how to improve engagement, and general advice.
Reason for taking part in this project	Barriers, motivation, and previous experience.
Thoughts on this project	Positive aspects, tips to improve the project, and project outreach.

#### Views on Social Media

Participants highlighted several advantages of social media, including “large outreach” (12 references), “convenience of getting things done from the comfort of their homes” (10 references), and “accessibility to opportunities” in fields of interest (7 references). Participants also noted that social media is beneficial for “finding information” (8 references), advertising and fundraising (5 references), and expanding audience and outreach (7 references). However, several participants shared their concerns about social media. A total of 7 participants referenced “misinformation” as a key threat in social media that should be taken seriously. Some participants expanded to explain the consequences of inaccurate information, such as anxiety (5 references), confusion (3 references), or a false sense of reassurance (3 references). A total of 4 participants commented on the potential of “wasting time” on social media.

#### Advice on Making the Most of Social Media

The most common themes were being aware of “audience’s demographics,” “sharing on more than one social media platform” (5 references), “collaborating with well-known organizations” (3 references), and “linking the videos to reliable web pages with more information” (2 references). There were also comments on how to maximize positive outcomes and minimize negative ones when using social media generally, including “the importance of monitoring and limiting the unproductive time spent on it” (9 references) and the importance of verifying any information for an unknown source (7 references).

#### Thoughts on the Project

Participants mentioned “non-restricting participation criteria,” “convenience” (n=8), and “ability to record videos from home with a pre-written script” (6 references), which made it a lot easier to participate. In addition, 3 participants mentioned the time needed to memorize the scripts (3 references), the need to step out of their comfort zone (1 reference), and the dates the volunteers were recruited (1 reference), made their participation challenging.

Tips to improve the project included having a “meetup with other volunteers” (6 references), increasing the “variety of video formats” submitted (4 references), and “extending the period that students had to submit their project” (2 references). Participants believed the project is beneficial for people “impacted by the respective conditions” (16 references), “healthcare professionals” (7 references), and “medical students” (6 references). All participants also stated that they believe this project design is “sustainable” for the long-term (n=8).

### Impact Evaluation

#### Quantitative Assessment

The 21 videos generated a total of 108,210 views across platforms, with Twitter and TikTok generating the most views (n=47,342), followed by Instagram (n=13,207), and YouTube (n=13,773), as of September 19, 2023, as summarized in Table 2 below. The highest number of views and shares on a single video were found in TikTok (4327 views, 57 shares), followed by Twitter (4176 views, 53 shares), whereas YouTube had the lowest views and shares (738 and 0, respectively). TikTok was also the platform with the highest number of likes, saves or bookmarks, and likes and saves for a single video, compared with the other platforms. The videos viewed and shared the most across all platforms were those about thyroid nodules, thyroid and pregnancy, and PCOS and thyroid. Between March 2, 2023, and March 26, 2023, the project’s social media profiles had an overall increase of 259 subscribers or followers (64.8 on average), with 178 new users subscribed on YouTube, followed by TikTok (n=31), Twitter (n=28), and Instagram (n=22). TikTok was the best-performing platform for new users and subscribers.

**Table 2:** Summary of data from the videos posted across all social media platforms as quantified on May 25, 2023.

Indicators and platforms	TikTok	Instagram	YouTube	Twitter	Total (average, SD)
Total number of views	47,342	13,207	13,773	28,888	108,210 (27,052, 13,935.80)
Highest number of views for a single video	12,140	1440	4118	4252	21,950 (5488, 4001.25)
Total likes	611	124	138	94	967 (241.5, 213.78)
Most likes for a single video	114	20	33	14	211 (53, 40.28)
Total shares	82	20	0	53	155 (39, 31.33)
Highest number of shares for a single video	24	3	0	9	36 (9, 9.25)
Total number of saves or bookmarks of videos	101	9	0	0	110 (27.5, 42.59)
Highest number of saves for a single video	29	3	—^a^	—	32 (16, 13)
Change in number of followers or subscribers (between March 2023 and September 2023)	+38	+22	+356	+128	+544 (136, 133.29)
% change in the number of followers or subscribers (between March 2023 and May 2023)	+950%	+8%	+32%	+26%	—

^a^Not applicable.

#### Qualitative Evaluation of Comments

A total of 38 comments were posted across all platforms as of September 19, 2023. Out of these, 17 were either emoji-based comments or commended the video without providing further context. In addition, 21 comments were further analyzed. Of these, 8 included praises for the project team, and 3 came from a spammer who promoted their services maliciously and spread misinformation. Furthermore, 5 asked for advice and further elaboration on conditions discussed in the videos. In total, 3 viewers shared their journey, specifically fears linked to the diagnosis and dissatisfaction with the diagnosis process. One of the viewers mentioned it provided helpful academic context as they had an assignment on a related topic. Another user shared that they had no idea about these conditions and thanked the team for publishing this information.

## Discussion

### Principal Findings

This report describes our experience generating, creating, disseminating, and evaluating evidence-based, peer-reviewed short social media videos. In this experience, we focused on PCOS and thyroid diseases based on our collective expertise. To our knowledge, this is the first study that describes the development and evaluation of videos for multiple social media platforms and discusses the abovementioned topics. Previous research has focused on other conditions (eg, low back pain) or specific surgical procedures (eg, laparoscopic hysterectomy) without guiding medical content creation [13,14].

Several studies have evaluated the content of short videos published on social media networks, highlighting unsatisfactory quality videos and warning about blind reliance on online content [24-26]. These studies acknowledged misinformation and invalidity as major factors compromising video content and negatively influencing the audience. This, paired with the acknowledgment of the increase in reliance on social media for medical information, flags the importance of starting evidence-based awareness campaigns and medical education initiatives for the masses. While previous researchers assessed the quality of online short videos across multiple social media platforms, none directly tackled the issues or attempted to generate evidence-based short videos.

This study builds on the KTA framework and focuses on knowledge dissemination [20]. After noting the rise in popularity and the increase in public reliance on short-video platforms as a source of medical information and acknowledging the issues linked to it, our study decided to use the short-video social media platforms to translate accurate knowledge and evidence-based information to the public. We adapted the information to be translated in lay terms and duration suitable for such platforms. Awareness of the social media algorithms, such as hashtags in promoting videos and attracting the right audience, allowed us to minimize barriers and facilitate knowledge transmission. Assessment of post video release of engagement and content creator feedback helped select and highlight obstacles and facilitators to this process of video dissemination. This methodology can be used to tailor further videos in other specialties. The dissemination of correct information could positively impact health behaviors, encouraging prompt diagnosis, preventing disease prognosis, and allowing an early management plan to be followed. Using the framework for content creation in the project is a way of standardizing and easing this process [27].

Involving medical professionals as content creators on social media brings a unique blend of credibility and educational value to combat misinformation and build trust in public health care. Their expertise allows for accurate and impactful messaging, addressing complex health topics, and debunking myths effectively. Time constraints, oversimplification, and regulatory considerations are some of the challenges that need to be addressed. Alongside creating misinformation-debunking videos, leveraging medical professionals’ knowledge can significantly contribute to raising awareness, promoting healthy behaviors, and fostering a culture of informed decision-making among the public.

### Process Evaluation

Responses from participants suggest that this project benefits health care professionals, patients, and the public. The experience gained from working on this video series has been noted to help medical students and health care practitioners at different levels of training. It also harnesses their skills and knowledge to ensure that the patient and public get the most up-to-date and accurate information while allowing them to ask questions and suggest further video topics. While some participants emphasized the potential for reaching wider audiences and fostering connections, others expressed concerns about social media engagement’s competitive nature and potential negative impacts. These varying viewpoints indicate the complex interplay between the benefits and drawbacks of using social media platforms for content dissemination. Our thematic analysis underscores the multifaceted nature of content creators’ motivations, which extend beyond monetary incentives and highlight the intrinsic rewards associated with engaging in creative endeavors. Participants’ positive feedback and constructive suggestions indicate ownership and investment in the project’s success. These insights contribute to the academic discourse on digital content creation and offer practical implications for educators, marketers, and content creators aiming to navigate the dynamic landscape of social media platforms. This study’s findings underscore the need for a holistic approach to understanding content creators’ motivations and behaviors within the ever-evolving realm of digital media.

### Impact Evaluation

We noticed an incremental engagement with our videos on all social media platforms over time. The videos were shared and saved across all platforms, implying their circulation as the audience uses multiple social media platforms. All social media platforms also witnessed an increase in followers, indicating that the public found our video series helpful. The varied number of views received underscores the diverse audiences on different platforms and highlights the potential for content to resonate strongly within specific communities. TikTok’s exceptional performance in likes, shares, and saves further highlights its potential for viral content dissemination and user interaction while aligning the content per platform-specific trends, formats, and audience preferences.

Our qualitative analysis highlights the dual nature of online engagement, from spammers spreading misinformation to genuine users sharing personal experiences and expressing gratitude. The presence of spammers underscores the need for robust moderation mechanisms to ensure a safe and accurate information-sharing environment with proactive community management. The inquiries and comments seeking further information about specific medical conditions suggest an avenue for generating additional content that addresses viewers’ questions and unmet needs. We highlight the diverse nature of engagement on different platforms and reveal the potential for meaningful engagement, education, and community-building through project-specific content. These findings offer insights for future content creation, platform selection, and audience engagement strategies, emphasizing the importance of tailoring content to different platform dynamics and user expectations.

### Recommendations

In the absence of evidence-based guidelines to generate medical social media content, this study allowed us to formulate a set of recommendations, which we summarized in a checklist that can also be used in further studies (Table 3).

**Table 3:** Recommendations for creating videos on medical topics for dissemination in the public domain.

Recommendation	Rationale
Recommendation 1: involving all stakeholders, including health care professionals at all levels, students, patients, and the public.	All stakeholders mutually benefited from the diverse perspectives to deliver easily understandable content for a large audience.
Recommendation 2: using a variety of video formats (ie, interview-style videos, role play, or Q&A^a^ format).	Content creators explained that they believe this will prove more engaging and, hence, be more appealing to participants.
Recommendation 3: linking the videos to information pages.	This will join between both the recent reliance on social media videos and the use of accurate research-based findings.
Recommendation 4: signposting the audience to relevant peer-reviewed studies or pages or organizations.	Misinformation spreads from the inability to distinguish reliable and nonreliable information. By signposting credible organizations, you point the audience in the right direction.
Recommendation 5: allowing the content creators to meet beforehand and familiarize themselves with one another and the project leads.	Volunteers suggested that familiarizing themselves with their colleagues would have helped build context and reach out for feedback on their respective videos to optimize video quality.

^a^Q&A: question and answer.

### Strengths and Limitations

The strengths of our initiative include using a collaborative approach of involving multiple stakeholders to generate credible peer-reviewed videos with ease of understanding. Feedback from viewers and volunteers who made the video provides essential insights into the public’s unmet needs and the importance of disseminating reliable information to debunk misinformation. Furthermore, according to Bloom’s Taxonomy [28], allowing students to engage with patients and health care professionals, analyze and evaluate video content, and generate these videos constitutes the highest form of learning. On the other hand, our study was limited by the small sample size of volunteers, videos, and total viewers. The sample size of content creators, consisting primarily of medical students and junior doctors, may not represent a diverse range of perspectives or experiences in content creation. This could limit the generalizability of findings regarding motivations, challenges faced, and overall experiences of content creation on social media platforms. In addition, as the results of online social media platforms are dynamic over time, similar studies conducted at different times or regions may yield different results. While this study focused on creating and disseminating peer-reviewed videos on PCOS and thyroid conditions, it may not encompass the full spectrum of health-related topics relevant to broader public health concerns. Furthermore, the evaluation period of 2 months for video engagement and audience outreach may provide only a snapshot of the long-term impact and sustainability of such initiatives. Future research could address these limitations by including a more diverse range of content creators, expanding the scope of health topics covered, and conducting longer-term evaluations to assess maintained audience engagement and behavior change. Obtaining detailed demographic and socioeconomic profiles of both volunteer and nonvolunteer groups will offer invaluable insights into the barriers hindering participation. These data will not only aid researchers in understanding the dynamics influencing volunteerism but also furnish crucial information for the design and implementation of future projects. Furthermore, uncovering these barriers could shed light on broader societal issues, potentially informing policies and interventions aimed at fostering greater community engagement and participation.

### Conclusion

Our study demonstrates how to codesign, disseminate, and evaluate evidence-based, peer-reviewed medical information for short videos to be distributed across social media platforms. This experience focused on PCOS and thyroid diseases and showed how social media could be used to increase awareness and tackle misinformation about these issues. In particular, social media videos can be used to engage the public and stimulate patients who might ask questions. This benefits both the viewers and the video creators, especially if they are medical students or junior doctors.

## Appendix

Multimedia Appendix 1 Public Engagement interview questions asked to volunteers who filmed the videos.

## Abbreviations

KTA: Knowledge-to-action
MeSH: Medical Subject Headings
PCOS: polycystic ovary syndrome
SIMBA CoMICs: Simulation via Instant Messaging for Bedside Application–Combined Medical Information Cines

## Data Availability

All data generated or analyzed during this study are included in this published article (and its supplementary information files). Further inquiries can be directed to the corresponding author.
