# Supplementary material for: A SIMBA CoMICs Initiative to Cocreating and Disseminating Evidence-Based, Peer-Reviewed Short Videos on Social Media: Mixed Methods Prospective Study
Source: JMIR Med Educ. 2024 Oct 30;10:e52924. doi: 10.2196/52924 (PMC11561432; doi:10.2196/52924)
Supplement: Multimedia Appendix 1 [file mededu_v10i1e52924_app1.docx]

Public Engagement interview questions asked to volunteers who filmed the videos.

**Experience before**

1. What do you think are effective ways to approach and engage the communities globally?
2. Do you have any prior experience using social media to inform viewers? Do you have any previous experience working on similar projects?
3. What is your experience with social media? How do you find it?

**Motivation**

1. Why did you decide to engage with this project? What are the motivations and barriers to participate?
2. Do you think this project is beneficial and sustainable? Would you recommend the videos we created to others?

**Experience working on this project**

1. In this project, what do you think went well? What could have gone better?
2. What do you think are the key factors impacting engagement on social media?
3. What improvements can be further made to increase public engagement in the field of endocrinology?
4. What can we do better to improve our engagement with you and others with whom you worked?
5. Would you like to add anything else?

**Interested in future work.**

1. If we conduct similar projects, would you be interested in taking part?
